# Fully-Automatic Synapse Prediction and Validation on a Large Data Set

**DOI:** 10.3389/fncir.2018.00087

**Published:** 2018-10-29

**Authors:** Gary B. Huang, Louis K. Scheffer, Stephen M. Plaza

**Affiliations:** Janelia Research Campus, Howard Hughes Medical Institute, Ashburn, VA, United States

**Keywords:** connectomics, synapse prediction, deep learning, quantitative evaluation, *Drosophila*

## Abstract

Extracting a connectome from an electron microscopy (EM) data set requires identification of neurons and determination of connections (synapses) between neurons. As manual extraction of this information is very time-consuming, there has been extensive research efforts to automatically segment the neurons to help guide and eventually replace manual tracing. Until recently, there has been comparatively little research on automatic detection of the actual synapses between neurons. This discrepancy can, in part, be attributed to several factors: obtaining neuronal shapes is a prerequisite for the first step in extracting a connectome, manual tracing is much more time-consuming than annotating synapses, and neuronal contact area can be used as a proxy for synapses in determining connections. However, recent research has demonstrated that contact area alone is not a sufficient predictor of a synaptic connection. Moreover, as segmentation improved, we observed that synapse annotation consumes a more significant fraction of overall reconstruction time (upwards of 50% of total effort). This ratio will only get worse as segmentation improves, gating the overall possible speed-up. Therefore, we address this problem by developing algorithms that automatically detect presynaptic neurons and their postsynaptic partners. In particular, presynaptic structures are detected using a U-Net convolutional neural network (CNN), and postsynaptic partners are detected using a multilayer perceptron (MLP) with features conditioned on the local segmentation. This work is novel because it requires minimal amount of training, leverages advances in image segmentation directly, and provides a complete solution for polyadic synapse detection. We further introduce novel metrics to evaluate our algorithm on connectomes of meaningful size. When applied to the output of our method on EM data from *Drosphila*, these metrics demonstrate that a completely automatic prediction can be used to effectively characterize most of the connectivity correctly.

## 1. Introduction

**Figure 1:**
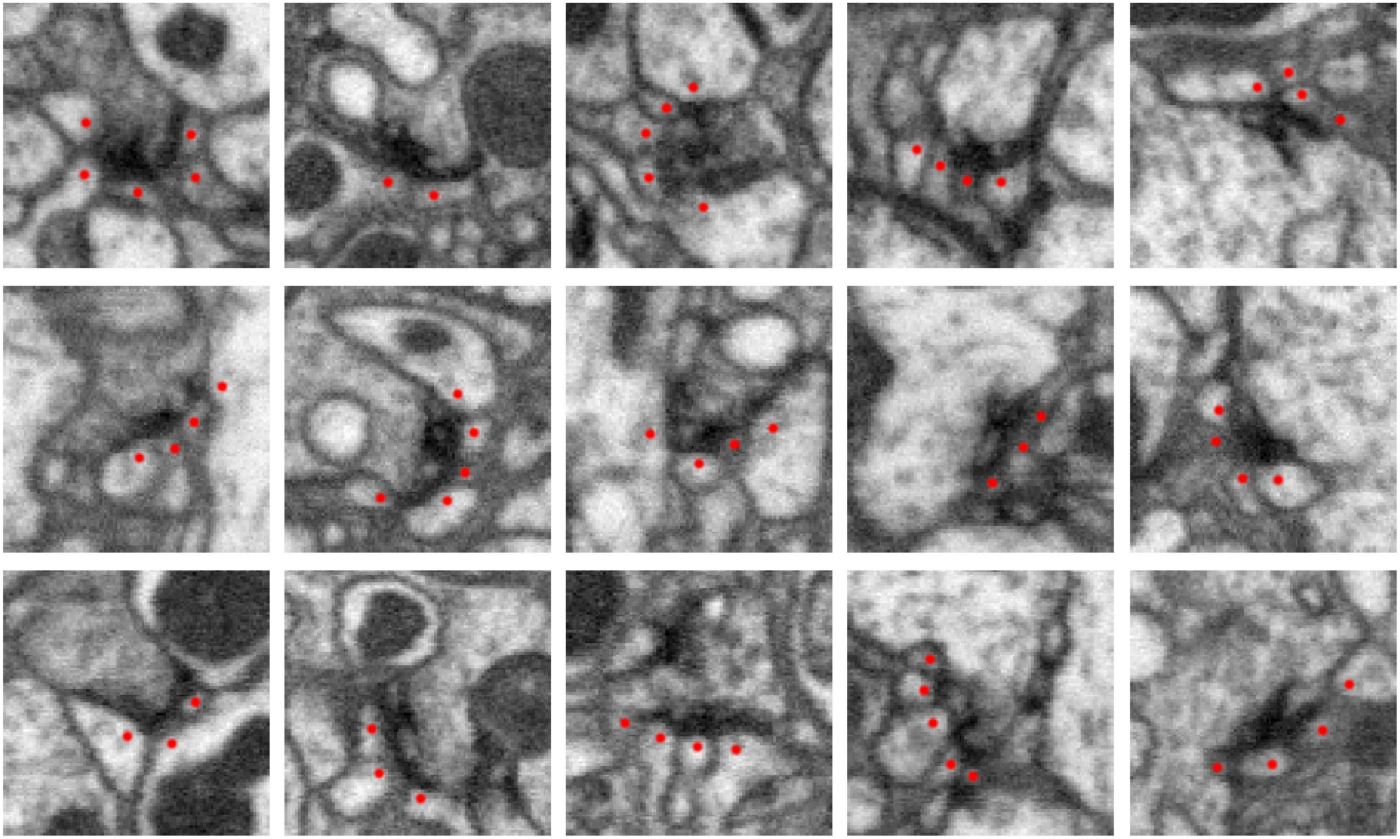
Five examples of synapses in the *Drosophila* optic lobe (columns). Rows show three orthogonal views (*xy*, *yz*, *xz* slices) of each synapse. The presynaptic structure, referred to as a T-bar because of its shape, is centered in each image. Red dots mark segments containing postsynaptic densities (PSDs) that partner with the T-bar. Each image slice captures 1 μm^2^ of data.

High-resolution EM imaging allows one to identify synapses, such as those shown in Figure 1 below. In these examples, there is an electron dense region corresponding to the synapse at the pre-synaptic body. This consists of different transport apparatuses, such as vesicles, that abut the neuronal membrane. In a data set that contains numerous organelles of varying electron densities (i.e., imaging intensity) and neuronal membrane that intricately weaves throughout, identifying synapses can be challenging. When creating a connectome, an annotator will typically scan the data set or a traced neuron and manually identify and mark these sites. Even for organisms as small as a fruit fly, there are up to 100 million connections, making the process of manual annotation intractable.

**Figure 2:**
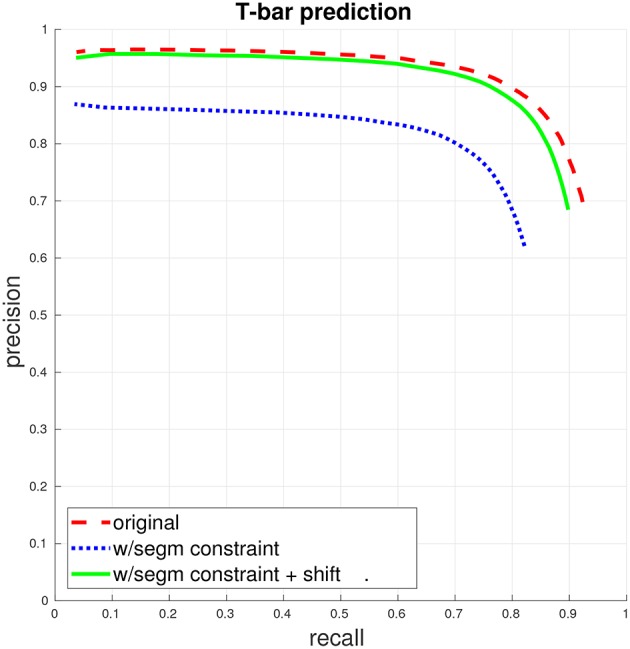
T-bar precision/recall. (This figure and subsequent figures are best viewed in color.) The dashed red curve indicates PR when the only constraint for a match between a predicted T-bar and a ground-truth T-bar is that the two locations fall within a specified distance from each other. The dotted blue curve gives PR when a match is further constrained to enforce that the predicted T-bar and ground-truth T-bar both fall within the same segment, in the ground-truth segmentation. Finally, the solid green curve gives PR with the segmentation constraint, if the predicted T-bar locations are first shifted slightly, away from potentially ambiguous regions. See the accompanying text and section 3.1 for more discussion.

Consequently, there have been recent research efforts to automate synapse detection using machine learning, which we discuss below in section 2. However, existing techniques for automated synapse detection have primarily been applied to detection in mammalian tissues. It is unclear, then, how well such approaches would translate when they are applied to synapse detection in *Drosophila* tissues. In contrast to synapses in the mammalian brain, which are predominantly monadic, involving a single presynaptic site and postsynaptic site, synapses in *Drosophila* are mostly polyadic, involving multiple postsynaptic partners for a given presynaptic site (Cardona et al., 2010), as can be seen in the examples shown in Figure 2. Even when the pre-synaptic site is given, these neuronal processes are often difficult to segment, which makes identifying the post-synaptic partners nontrivial.

With the exception of Staffler et al. (2017), prior work on automated synapse identification, as discussed below in section 2, has also focused only on the detection problem in isolation, with performance evaluated at the individual synapse level. However, synapse detection is one step in a larger pipeline, whose final goal is the extraction of a connectome from the electron microscopy (EM) data. Therefore, we are interested not only in individual synapse detection accuracy but also in how synapse detection integrates into this larger system and how errors in individual steps in this system combine when evaluating the final produced connectome.

For instance, one straightforward method for reliably using automated synapse detections in an EM pipeline is as hints for manual annotation, as done by Plaza et al. (2014). By manually verifying detections, errors in the final connectome are minimized but at the expense of human effort and time. An alternative would be to simply accept all detected synapses above a certain confidence threshold, but there has been limited prior work on whether such a prediction would result in a meaningful connectome (Dorkenwald et al., 2017). In particular, many connections between neurons are formed from a large number of synaptic contacts, and, therefore, one might hope that automated algorithms are capable of faithfully reconstructing such high strength connections, but there has been limited experimental testing in this direction.

Moreover, extracting a connectome is also dependent upon automatic neuron segmentation. In addition to possibly being outright incorrect, a segmentation may also be noisy along a border. Both cases may potentially cause errors in the connectivity graph when combined with the automated synapse identification output.

To our knowledge, these questions of evaluating synapse detection in a larger context have only been investigated in the recent work by Staffler et al. (2017). They find that many synapse detection errors occur near errors in automated segmentation and that manually fixing these segmentation errors is sufficient to correct nearly half of the synapse detections. They also give a theoretical analysis of individual synapse accuracy vs. binary neuron-to-neuron connection accuracy, assuming a distribution of synapses per neuron pair estimated from paired recordings in rodent cerebral cortex; additionally, they compute synapse accuracy and neuron-to-neuron level accuracy on a sparse local cortical connectome.

Therefore, in this paper, we introduce algorithms that enable fully automatic synapse prediction and evaluate the results of the end to end process from the standpoint of the final produced connectome. Specifically, key contributions and results of our approach include the following:

1. an algorithm that generalizes well over a large data set of *Drosophila* tissue with minimal supervision required,
2. new metrics to better evaluate synapse prediction in realistic settings, and
3. empirical results analyzing the end to end accuracy of the proposed approach on a publicly available connectome data set (Takemura et al., 2015), demonstrating high performance and preservation of biological pathways, in particular, relative to a baseline using body-proximity as a proxy for synaptic contact.

## 2. Background

An automated approach for synapse identification in EM images using machine learning was first proposed by Kreshuk et al. (2011), who used a random forest (RF) classifier on hand-selected image features to detect synapses. In a subsequent study, Kreshuk et al. (2014) extend this method by applying graph cut on the synapse probabilities to obtain a segmentation of each putative synapse, extracting object-level features when the segmentation is given, and then applying a RF classifier to determine whether each segmented region is a synapse or not.

Becker et al. (2013) attempt to generate more informative features, by conditioning on the synaptic cleft, thereby, allowing features to be extracted from consistent spatial locations relative to the putative synapse. These features are then used as input for AdaBoost for synapse detection. Staffler et al. (2017) extend this by conditioning on presynaptic and postsynaptic regions separately, and use extracted features from these regions as input for LogitBoost for synapse detection, yielding improved results.

Jagadeesh et al. (2013) consider the problem of large-scale synapse detection in a large image volume. They first use a fast interest point detector based on image-thresholding to generate proposals for possible synapse locations. They then use feature descriptors hand-designed to extract information about relevant biological structures, namely vesicles, clefts, and ribbons. These features are used as input for a support vector machine (SVM) or a multiple kernel learner for patch-based synapse detection.

Biological preparation has also been considered as a means to aid automated synapse detection. Navlakha et al. (2013) apply a technique for the selective staining of synapses, leading to more pronounced opacity at synaptic sites, and leaving non-synaptic membranes unstained. They propose a high-throughput method for automated detection by first filtering down to a candidate set of patches and then applying an SVM to classify each patch as synapse or non-synapse. While this technique can be used to compute statistics on synapses such as density, since the membranes are left unstained, it cannot be used in conjunction with segmentation and, therefore, cannot be directly used for extracting a connectome.

More recently, Roncal et al. (2015) also consider large-scale synapse detection, presenting two different techniques. They propose a fast RF classifier using hand-selected features, including a filter designed for vesicle detection. This RF classifier yields results that are similar to the results of Becker et al. (2013) but with approximately half the run-time. They also propose a deep learning classifier for synapse detection, which yields results that are superior to the fast RF classifier but is approximately two orders of magnitude slower. Dorkenwald et al. (2017) also give a deep learning multiclass CNN for detecting synapses along with vesicle clouds and mitochondria and report improved results over Roncal et al. (2015).

The above approaches were evaluated on synapse detection in mammalian tissues, assuming a single postsynaptic site for each presynaptic site. Several approaches also make additional assumptions on the data, such as being able to reliably identify the synaptic cleft to extract spatially consistent features (Becker et al., 2013) or having feature descriptors hand-tuned for particular biological structures (Jagadeesh et al., 2013).

While manual annotation of synapses has been performed for sparse EM reconstructions (Zheng et al., 2018) and software tools have been created to facilitate manual synapse annotation (Boergens et al., 2017), a scalable alternative to facilitate reconstruction of larger connectomes is to make use of automated methods within a semi-manual workflow. For example, the method of Kreshuk et al. (2011) was adapted for presynaptic site detection by Plaza et al. (2014), where human proofreaders subsequently verified or rejected each automated detection, but the labeling of postsynaptic partners was performed manually with no automated guidance. Takemura et al. (2017) also took a similar approach, using the method of Huang and Plaza (2014) to generate presynaptic site proposals, with postsynaptic partner identification again being performed manually.

As mentioned in the introduction, synapse detection in *Drosophila* can be more challenging, owing to the polyadic nature of such synapses, where presynaptic sites have multiple postsynaptic partners and where postsynaptic processes can often be small and difficult to segment. To address this difficulty in *Drosophila* synapse detection, Kreshuk et al. (2015) specifically studied the problem of synaptic partner assignment. Conditioned on ground-truth neuron segmentation and synapse detection, they formulate a pairwise graphical model wherein nodes of the model, *P*_*ij*_, represent possible assignments between two neurons *i, j* at a putative synapse, for example, neuron *i* is presynaptic and neuron *j* is postsynaptic. Edges in the model, connecting *P*_*ij*_ and *P*_*ik*_, encode biological priors on triplets of neurons *i, j, k* at a putative synapse, such as a preference for a one-to-many connection pattern over one-to-one. In a recent study, Heinrich et al. (2018) propose a deep learning CNN model for synaptic cleft segmentation in *Drosophila*, achieving state of the art results (evaluated on the CREMI challenge data set). With its specific focus on voxel-wise identification of the synaptic cleft, this work can be distinguished from ours in that we attempt to directly predict synaptic connectivity, which would require a nontrivial subsequent step conditioned on the cleft prediction output (for instance, by applying the method of Kreshuk et al. (2015).

In this study, we propose a complete system for automated synapse detection, capable of handling polyadic synapses as found in *Drosophila*. Our system uses a weakly-supervised deep learning approach and takes the simple point-wise annotations of presynaptic and postsynaptic sites as training data and, therefore, can be applied to new data sets with relatively minimal supervision. By comparison, existing methods as discussed above, which require hand-designed features to extract high-level information such as vesicles and ribbons, may not be appropriate for new data sets or may require significant manual effort to tune or redesign the feature descriptors. In contrast to Kreshuk et al. (2015), we evaluate our system on completely automated, noisy segmentation. Although our overall system was designed for synapse detection in *Drosophila*, in section 6, we discuss how the elements of our approach could be adapted for other domains such as mammalian tissue.

## 3. Automated synapse detection

Our system for automated synapse detection proceeds in two distinct steps. First, independent of any segmentation, we apply a classifier to automatically identify presynaptic sites in *Drosophila*, which are often referred to as T-bars, because of their T-like shape, formed by a pedestal and platform structure. Next, conditioned on predicted T-bar locations and a segmentation, we apply a second classifier to predict partnering postsynaptic densities (PSDs) for the identified T-bars.

Owing to its distinct structure, we focus on first predicting T-bars in isolation, independent of both segmentation and PSD prediction, and delay the problem of determining the potentially multiple PSD partners until after segmentation, as PSDs are typically more ambiguous and difficult to identify. We note that this approach of splitting T-bar and PSD prediction into separate steps, with PSD prediction aided by segmentation, has also been employed for manual synapse detection (Plaza et al., 2014).

We describe each step in our pipeline in more detail in the next two sections. We have also released source code that implements the proposed methods^1^.

### 3.1. Presynaptic T-bar identification algorithm

The first step in our automated synapse detection pipeline is to detect the presynaptic T-bar sites. Examples of T-bars can be seen in Figure 1.

For automated T-bar detection, we follow the approach described in Huang and Plaza (2014), except that we update the voxel-wise classifier to be a 3D U-Net CNN (Ronneberger et al., 2015). We give an overview of our approach here; for more details, see Huang and Plaza's paper (2014).

Unlike the problem of image segmentation, which is naturally framed as a voxel-wise prediction problem (at each voxel, predict whether that voxel belongs to a cell boundary or not), T-bar detection is an object detection problem, which we formulate as predicting, for each T-bar, a point annotation, specifying the spatial coordinates of the center of the T-bar. To generate voxel-wise training data for the U-Net, we simply consider any voxels within a certain radius of a T-bar point annotation to be a positive example and all other voxels to be negative examples. We find that the U-Net is able to successfully learn from this simple training data, allowing for less manual supervision effort relative to methods and tasks that require dense labeling. Our specific U-Net model consists of layers of convolution with 3^3^ voxel filters and two downsampling and two upsampling layers, with a total receptive field size of 19^3^ voxels.

To generate final T-bar point predictions from the voxel-wise output of the U-Net, we spatially smoothen the voxel-wise predictions, selecting the voxels with highest confidence, and apply non-maxima suppression.

We make two notes concerning the evaluation of T-bar prediction, in the context of a larger connectomics pipeline. First, it is important to consider the precision/recall (PR) curve for the automated predictions. Different applications may have different misclassification costs, leading to different thresholds along different points of the PR curve. For instance, if very high fidelity is required, one may need to select a threshold for high recall, at the expensive of precision, whereas if the final goal is to determine strong connections in the connectome with some tolerance for small errors, the optimal threshold may be to select for the PR break-even point.

Second, T-bar prediction accuracy can be computed by necessitating for instance, that predicted T-bars be within a specified distance of a ground-truth T-bar to be counted as a correct match, as described by Huang and Plaza (2014). However, ultimately, the exact location of a T-bar annotation will be abstracted as one end point of an edge in a connectomic graph, indicating the presynaptic body. Therefore, the primary concern is that annotation be placed in the correct neuron. Thus, when a segmentation is available, T-bar prediction accuracy should be computed by further necessitating that the predicted T-bar falls within the same segment as the ground-truth T-bar.

Owing to this interaction with the segmentation when evaluating T-bar performance accuracy, it may be beneficial to post-process the T-bar predictions. For instance, we find that our T-bar detection often places the annotation in the distinctive dark T-like structure itself, which, owing to its dark intensity, can cause problems for automated segmentation. We, therefore, find a benefit in slightly shifting T-bar predictions within a small radius to the brightest intensity voxel, helping the annotation to be placed in a nonambiguous region relative to the segmentation.

### 3.2. Segmentation-aware postsynaptic partner identification

Once we automate T-bar predictions and a (possibly automated) segmentation, we condition on this information in order to predict the PSDs that partner with each T-bar. For a given T-bar, we can consider all nearby segments as potentially possessing a partner PSD. More precisely, we use the set of segments that have a non-empty intersection with a sphere of a given radius, centered at a given T-bar, as the candidate set of bodies that may be postsynaptic to the T-bar. We exclude the segment containing the T-bar itself and, therefore, make no attempt at predicting autapses. Additionally, we do not attempt to identify cases where a single T-bar makes multiple connections to the same postsynaptic body, and, thus, any such biological multiple-connections will at most be predicted as a single synapse.

With this setup, we have a binary classification problem, where for each T-bar and each candidate segment, we wish to determine if the candidate segment contains an actual PSD and, thus, forms a synapse with the T-bar. For classification, we use a multi-layer perceptron (MLP) with a single hidden layer consisting of 50 hidden units, trained using cross-entropy loss. To generate the feature representation, we estimate the interface of the synapse between the T-bar segment and candidate segment, by dilating both segments by varying amounts and letting the estimated interface to be the intersection. We then pool a set of simple image features over the interface, computing statistics such as size of the interface and image intensity within the interface (such as number of voxels with intensity lower than some given threshold), giving a total input feature dimensionality of 135.

One important consideration is that PSD prediction performance will depend on both the accuracy of the PSD predictor itself as well as the performance of the algorithm used to generate the segmentation. Therefore, it may be necessary to tune the PSD predictor with an awareness of the behavior of the segmentation algorithm. For instance, we found that dark intensity values such as those found at a boundary, as well as at T-bars, would often present difficulties for the segmentation algorithm. This ambiguity could lead to, for instance, small parts of the T-bar being incorrectly assigned to a neighboring segment. Although such localized errors would not have a large effect on the topology of the segmentation (in terms of Rand error, for example), they could have a large effect on the proposed feature representation and, hence, the PSD classifier. Therefore, we attempt to make the classifier more robust to such errors by ignoring the segmentation at voxels with such dark intensity values.

## 4. Metrics for evaluation

As discussed above, to properly evaluate automated synapse detection performance in the context of a larger pipeline, it is important to consider the full performance curve as the threshold of classifier confidence is varied. This allows for synapse prediction to be evaluated at the appropriate threshold for varying misclassification costs, which will depend on the final application that is being considered. One straightforward metric for evaluating detection at the individual synapse level is to produce a (PR) curve. Under the view of the connectome as a graph, with directed edges between nodes (representing neurons) defined by synapses, we can consider two variations for computing PR. First, we can view the connectome as a weighted graph and compute PR by considering each individual synapse as a ground-truth label that is to be predicted. Second, we can consider the connectome as an unweighted graph and compute PR by considering each edge (formed by any number of synapses between a pair of neurons) as a ground-truth label that is to be predicted.

The above methods for computing PR are two ways of dealing with the finding from connectomic studies that many connections between neurons consist of multiple synapses (Takemura et al., 2013, 2015). This multiplicity may be a weight on the synapse strength or may be a mechanism for robustness. In either case, a general assumption in many connectomic efforts is that important biological connections will have some multiplicity greater than one. Therefore, we would like to consider a range of metrics that will better reflect whether a set of automated synapse predictions is actually good enough for use in connectomic studies.

Computing PR with a weighted graph requires that the automated predictions match the ground-truth precisely in terms of strength, without any regard to topology. For example, predicting an edge of strength 7 for a ground-truth edge of strength 9 is equivalent to missing an edge of strength 2 (in terms of impact on total recall value), which may be inappropriate if we fail to care about precisely determining the multiplicity of strong connections. Computing PR with an unweighted graph, on the other hand, evaluates the automated predictions solely in terms of unweighted topology. Therefore, no penalty is incurred for not correctly determining multiplicity, but missing an edge of multiplicity 1 is equivalent to missing an edge of multiplicity 7.

One simple modification that can be made to the unweighted graph computation is to consider the unweighted graph produced by thresholding the edge weights by some value *t* (in both the predicted and ground-truth connectomes). For *t* = 1, we have the original unweighted PR; for *t* > 1, we focus only on stronger predicted and ground-truth edges, with multiplicity of at least *t*. We can also examine performance of a given classifier over different sets of curves as we vary this threshold *t*.

### 4.1. Asymmetric PR, connections added/missed

By thresholding the edge weights at some *t* > 1 and computing unweighted PR, we focus on the strong edge connections and ignore potentially noisy weak connections. However, there is still a strong boundary effect, where, for instance, a predicted edge of strength *t* − 1 for a corresponding ground-truth edge of strength *t* is counted as a false negative, the same as if the predicted edge strength had been simply zero. This harsh decision boundary may also be problematic from the standpoint of potential small errors in the manually annotated ground-truth. We would like a metric that focuses on identifying clear error cases in the automated predictions.

We, therefore, introduce an asymmetric variant of the above thresholded PR curve. Let the asymmetric *t*_1_, *t*_2_ thresholded PR curve (with *t*_1_ > *t*_2_) be defined as follows: consider the (weighted) ground-truth connectome graph *g* and the predicted graph *p* produced by applying some classifier threshold, and let *g*(*e*) be the weight of a given edge *e* in *g* and similarly for *p*(*e*). Recall is then computed as

(1) $$\frac{\sum_{e}\left[p(e)\geq t_{2}\wedge g(e)\geq t_{1}\right]}{\sum_{e}\left[g(e)\geq t_{1}\right]},$$

where the square Iverson brackets equate to 1 if the condition inside is true and 0 otherwise. In other words, the total set of positive ground-truth instances consists of all edges with ground-truth weight greater than *t*_1_, but the subset of true positives allows for edges with predicted weight greater than the smaller *t*_2_. Conversely, precision is computed as

(2) $$\frac{\sum_{e}\left[p(e)\geq t_{1}\wedge g(e)\geq t_{2}\right]}{\sum_{e}\left[g(e)\geq t_{1}\right]}.$$

Here, the total set of positive predicted instances consists of all edges with predicted weight greater than *t*_1_, but the subset of true positives allows for edges with ground-truth weight greater than the smaller *t*_2_.

From the above PR definitions, it can be seen that the asymmetric *t*_1_, *t*_2_ thresholded PR upper bounds the original symmetric thresholded PR at *t* = *t*_1_. This more lenient performance measure focuses on the more clear, egregious errors, where there is a strong edge in either the ground-truth or predicted connectome graph but a weak or no edge in the other graph. We can also report these types of errors directly as connections falsely added (false positives) and connections missed (false negatives). Let connections missed be the set of edges *e* such that *g*(*e*) ≥ *t*_1_ ∧ *p*(*e*) < *t*_2_. The number of connections missed is an unnormalized version of 1 − recall. Let connections added be the set of edges *e* such that *p*(*e*) ≥ *t*_1_ ∧ *g*(*e*) < *t*_2_. The number of connections added is an unnormalized version of 1 − precision. When plotting number of connections added vs. number of connections missed, we normalize these values by the number of edges in the ground-truth connectome after thresholding, that is, the number of edges *e* such that *g*(*e*) ≥ *t*_1_, to put curves with different values of *t*_1_ on the same scale.

By using asymmetric thresholded PR and connections added/missed, we can focus on strong error cases when comparing sets of predictions and be robust to small amounts of labeling noise. These error measures also more clearly indicate to what extent strong biological connections are being missed or falsely introduced through prediction.

## 5. Results

In this section, we present a case study of our proposed synapse detection system on data from the *Drosophila* optic lobe. The data set that we use comprises seven columns of the medulla, acquired using focused-ion beam milling scanning electron microscopy (FIB-SEM). The image data has a total volume of 40 × 40 × 80 μm, with an isotropic resolution of 10 nm per voxel. The manually annotated subset of the data that we use in this study consists of 27, 000 cubic microns and contains ~56,500 T-bars and ~336,500 PSDs. Our methods operate on the data at the original resolution. Additional details of the data can be found in the papers of Plaza et al. (2014) and Takemura et al. (2015), and the raw EM image data, FIB-25, is available online^2^.

We give results of the individual steps of our pipeline, full end to end results, results using the proposed error metrics focusing on clear error cases, comparison against a surface area contact baseline, and results in the context of preserving biological findings.

### 5.1. Performance of T-bar, PSD detectors

We first train a T-bar detector using the system described above in section 3.1, using the ground-truth annotations contained in two 520^3^ voxel subcubes of the total volume, containing a total of 325 T-bars. Figure 2 gives the precision/recall curve for the automated predictions over the entire data volume. The plot highlights two important points that were made in section 3.1: First, T-bar prediction accuracy should ideally be assessed within the context of segmentation and the final produced connectome graph, rather than only considering the distance between predicted and ground-truth T-bar locations. A predicted T-bar that is very close to a ground-truth T-bar, but placed in the wrong ground-truth segment, will lead to errors in the connectome graph. This is highlighted in the difference between the dashed red curve and the dotted blue curve. Consequently, second, the T-bar detector may need to be aware of the behavior of the corresponding segmentation algorithm. In our case, we found that simply shifting the predicted T-bar locations slightly, toward brighter image voxels, would move the predictions away from dark image regions that are more difficult or ambiguous for the segmentation algorithm and, therefore, improve performance when applying the segmentation constraint.

Next, we evaluate the performance of the PSD predictor. We consider performance both under the scenario in which we have access to the ground-truth segmentation and in which we only have access to a predicted, fully-automated segmentation. We first make a note about the “ground-truth segmentation.” This segmentation was produced by starting from an automated segmentation (separate and distinct from the fully-automated segmentation we use for synapse prediction) and manually proofreading the segmentation by applying merge and split operations as necessary. This ground-truth segmentation, therefore, aims to get the correct general topology, but it is not refined to the point of necessarily assigning a correct label at the voxel level, and additionally this segmentation may have orphan fragments that were not merged into larger bodies. One important consequence is that, when we compute performance using this ground-truth segmentation, we typically ignore all predictions that fall into such orphan fragments, defined as segments that contain neither a ground-truth T-bar nor a ground-truth PSD. In other words, predictions that fall into such fragments are not counted when computing precision. Additionally, we shift PSD point annotations using the same criteria as those used when shifting T-bar annotations as mentioned above.

**Figure 3:**
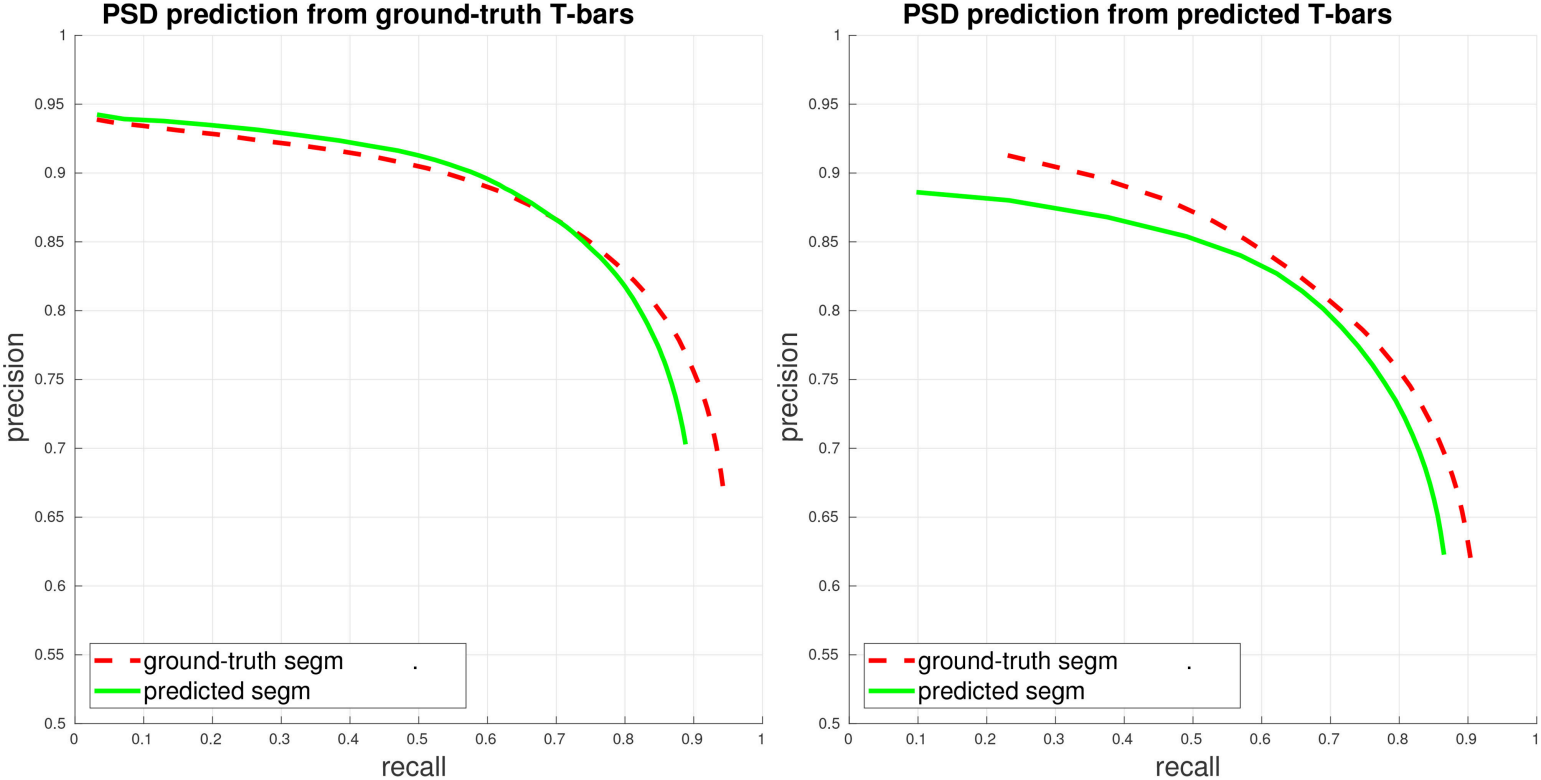
PSD precision/recall, where each PSD is considered separately (weighted view of connectome graph, see section 4). Performance is computed both when access to the ground-truth segmentation is available during PSD detection (ground-truth/gt segm), and when only the fully-automated, predicted segmentation is available during PSD detection (predicted/pd segm). **(Left)** Plot of PSD prediction performance in isolation, using ground-truth T-bar locations. **(Right)** Plot of end-to-end performance, using predicted T-bar locations.

We first evaluate PSD prediction assuming that we have access to ground-truth T-bar locations, in order to evaluate the performance of the PSD detector on its own. This performance is given in the left plot of Figure 3. Next, we evaluate PSD prediction using predicted T-bar locations (using a conservative threshold on the T-bar confidence scores, aimed at achieving a high recall of 0.9). We compute precision/recall considering each PSD separately, corresponding to a weighted view of the connectome graph. Importantly, we note that although performance is best when the ground-truth segmentation is available during PSD prediction, our PSD predictor is still able to achieve close performance using the automated, predicted segmentation.

### 5.2. End to end synapse performance and comparison

We now move from evaluating each of the detectors in isolation to giving a full end to end evaluation of our synapse detection pipeline, with respect to the final generated connectome. As determining an acceptable prediction accuracy is difficult without considering the particular connectomics application domain, we present a range of performance curves using our proposed error metrics. Additionally, we compare against a baseline using neuronal-body proximity/contact as a proxy for synaptic contact. For this baseline, we use the *ground-truth* segmentation. We randomly sample points at boundaries between ground-truth segments and then randomly select the direction of the synapse (presynaptic and postsynaptic bodies). For this proximity-based comparison, we also compute precision/recall using an undirected view of the connectome graphs, thereby, allowing for matches even if the predicted direction of synapse was incorrect.

**Figure 4:**
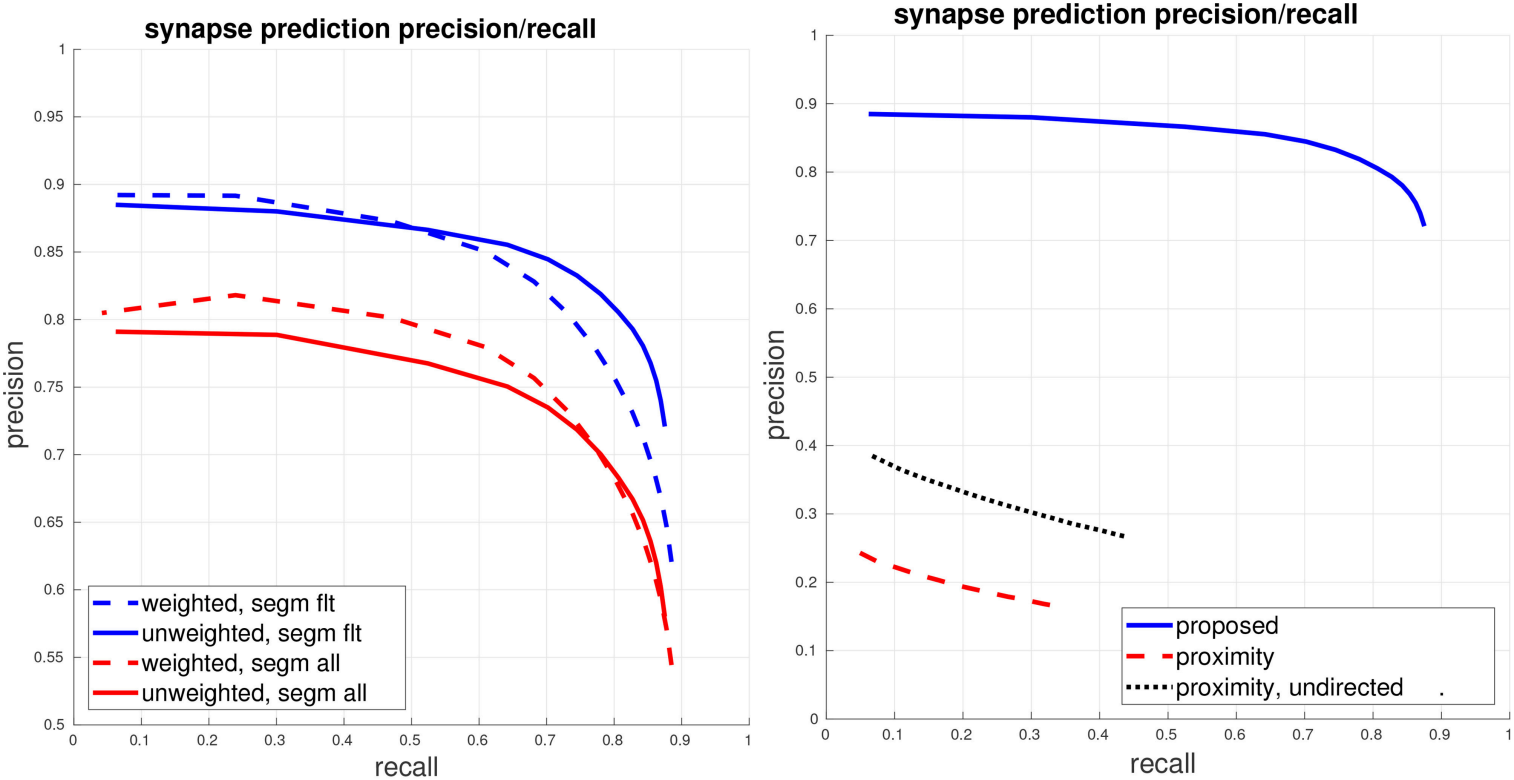
Global connectome graph precision/recall. **(Left)** The top blue curves show PR of the connectome graph, with the dashed curve computing PR using a weighted view of the graph edges and the solid curve computing PR using a unweighted binary view of the graph edges. These curves are computed using the filtered set of bodies in the ground-truth segmentation, as described in section 5.1. For reference, the bottom red curves show weighted and unweighted PR if all bodies (adding in orphan segments) are considered. **(Right)** Comparison against the baseline of using ground-truth body-proximity as a proxy for synaptic contact. All curves show unweighted PR on the filtered set of bodies.

The left plot of Figure 4 gives the PR of our proposed system, using the fully-automated predicted segmentation. We fix a conservative threshold for T-bars, accepting all T-bars above this threshold, and vary the threshold for the PSD detector to generate PR curves, under both a weighted and unweighted view of the connectome graph edges. The right plot shows a comparison against the baseline using body-proximity as a proxy for synaptic contact. Even after using the ground-truth segmentation and computing the undirected edge PR this baseline performs much worse.

**Figure 5:**
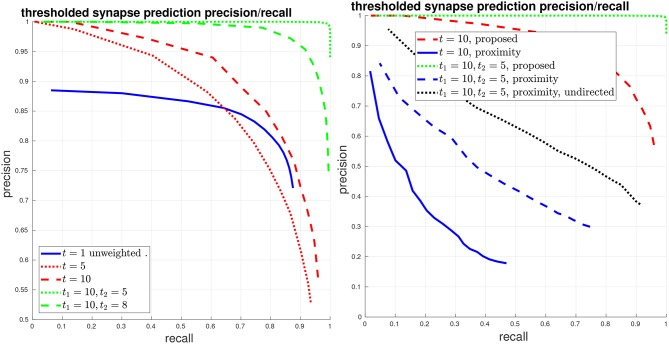
Thresholded global connectome graph precision/recall. **(Left)** The solid blue curve shows the unweighted PR from the previous figure, which is equivalent to a threshold of *t* = 1. The red curves give symmetric thresholded PR at *t* = 5, 10. The green curves show asymmetric thresholded PR at *t*_1_ = 10, *t*_2_ = 5, 8. **(Right)** Comparison against the baseline using body-proximity. Focusing on strong error cases shows that while the proposed method only makes a few mistakes at *t*_1_ = 10, *t*_2_ = 5, the body-proximity baseline still performs comparatively poorly.

Next, we evaluate synapse detection performance using our proposed variants to PR, as shown in Figure 5. We give curves when thresholding the edges at different values *t*, that is, a (unweighted) edge is preserved in the connectome graph if the original edge weight is greater than *t*. If *t* = 1, then the curve is equivalent to the above unweighted graph PR. We also give curves using our proposed asymmetric thresholded *t*_1_, *t*_2_ PR. We again compare with the baseline of using body-proximity.

**Figure 6:**
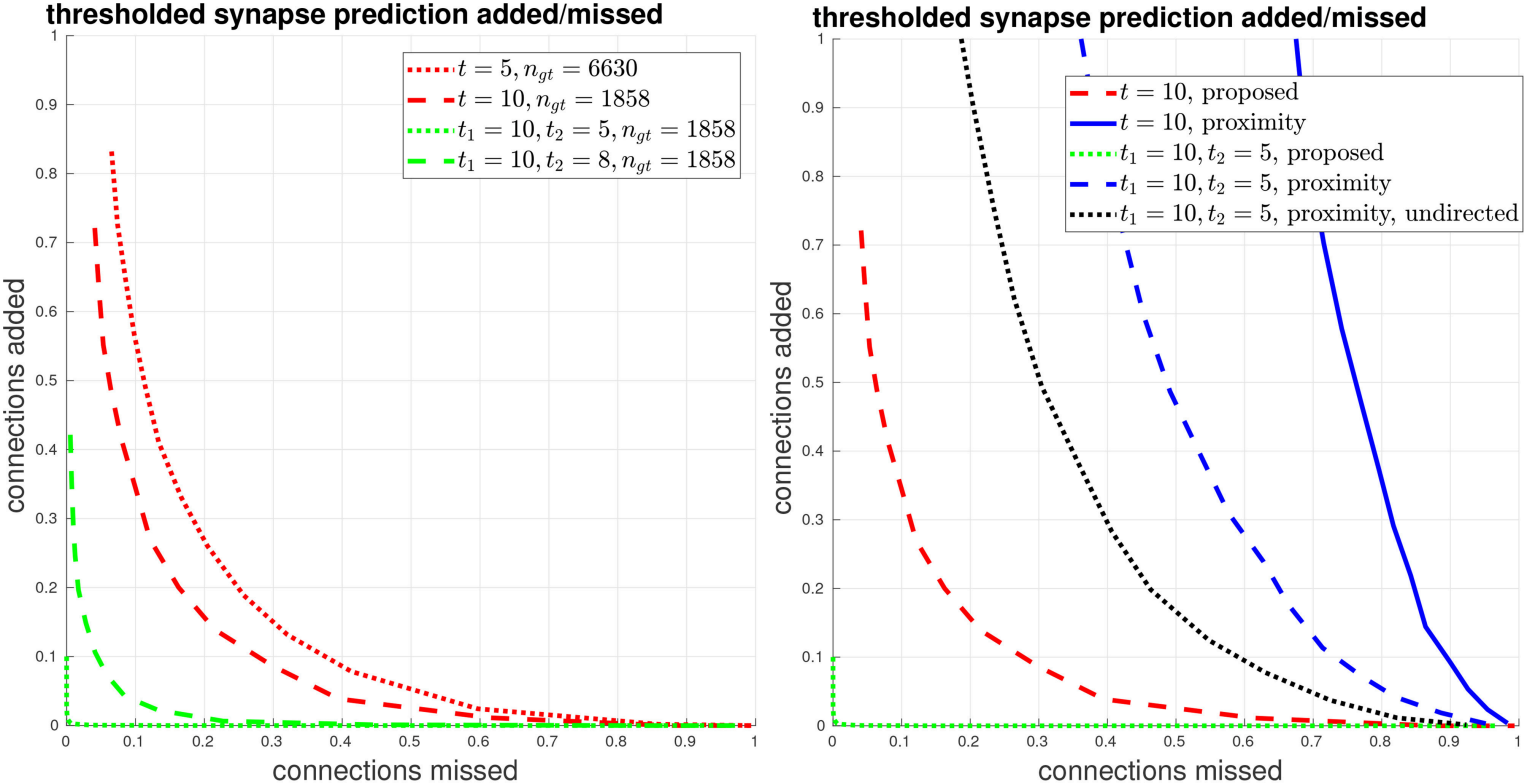
Thresholded global connectome graph connections added/missed. **(Left)** The curves show errors in terms of connections that were added and missed, using the same thresholds as the PR curves in Figure 5. **(Right)** Comparison against the baseline using ground-truth body-proximity, both directed and undirected edges.

We give another view of synapse detection performance, using our metrics of connections strongly added and missed, in Figure 6. For the case of thresholding with *t*_1_ = 10, *t*_2_ = 5, we have a total of about 2000 edges in the ground-truth connectome with a weight of at least *t*_1_ = 10. Using our proposed system, we can recover more than 99% of these edges (less than 1% connections missed) while introducing less than 1% falsely added connections. By comparison, from the right plot in Figure 6, we can see that by using body-proximity as a proxy for synaptic connection, when thresholding by *t*_1_ = 10, *t*_2_ = 5 and considering the directed graph, the normalized number of connections added and missed is approximately 50/50%. Therefore, even with this error metric that focuses on clear, unambiguous errors, this baseline approach is missing half the ground-truth connections and adding in approximately the same number of false connections.

**Figure 7:**
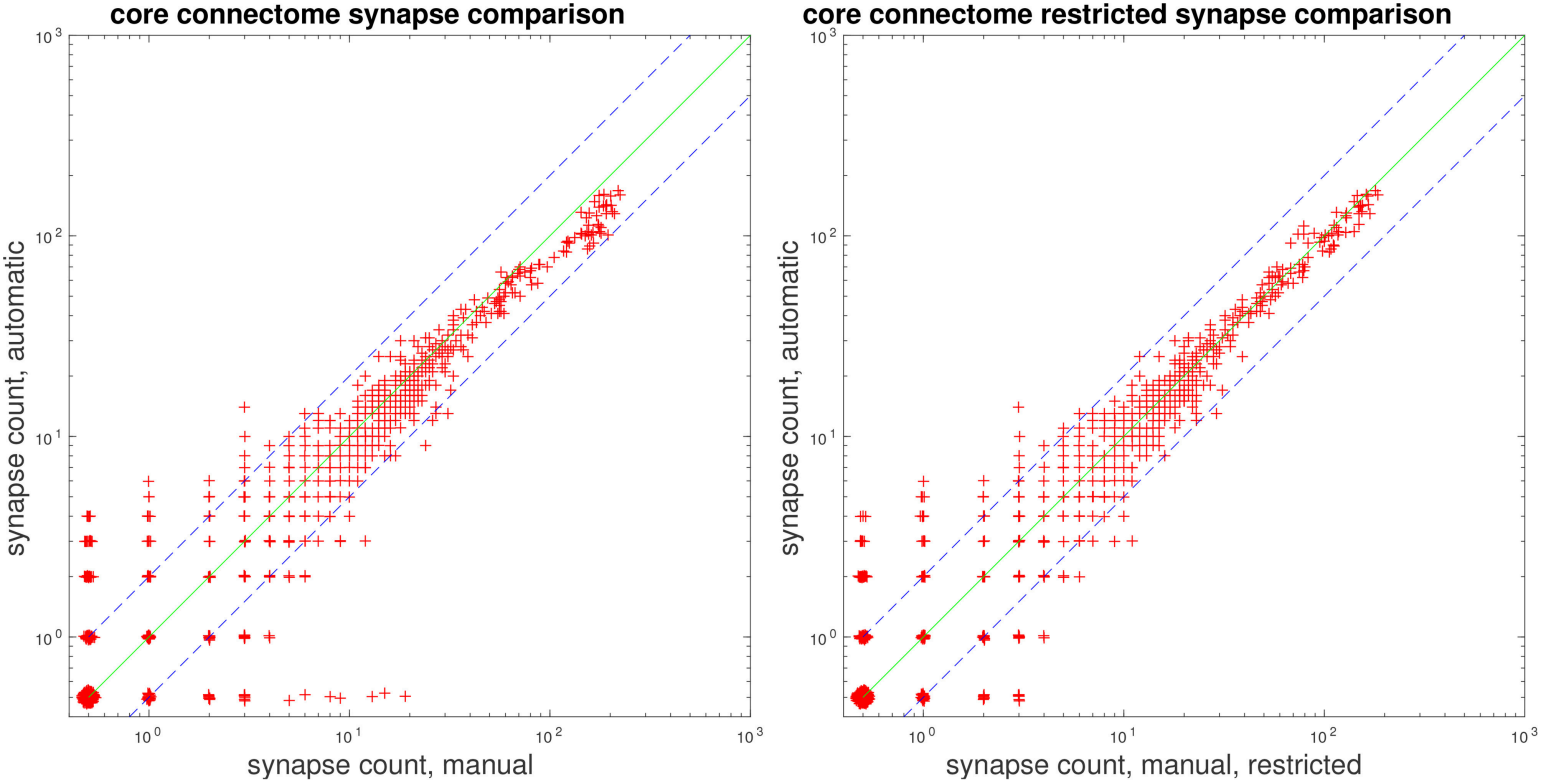
Comparison of manual and automatic synapse counts, where each point indicates the counts for an edge in the connectome. Edge weights of 0 have been shifted to 0.5 to appear on the log plot. Dashed lines indicate *y* = 2*x* and $y=\frac{1}{2}x$. **(Left)** Comparison with full ground-truth connectome. **(Right)** Comparison after removing ground-truth autapses and collapsing multiple connections from one T-bar to the same postsynaptic body.

Lastly, we present plots comparing automatic vs. manual synapse counts when restricting edges to a core set of bodies and connectomes, used in a study by Takemura et al. (2015). Figure 7 gives scatter plots, where each point gives the automated and manual synapse count for an edge in the connectome. As mentioned above in section 3.2, our proposed system has limitations in that it does not attempt to predict autapses and predicts at most one connection from a T-bar to a given postsynaptic body. Therefore, we also give a comparison of automatic vs. manual counts, shown to the right in Figure 7, after removing autapses and collapsing multiple connections from a single T-bar to the same postsynaptic body.

We note that, for strong edges with a synapse count of 30 or above, our automated predictions fall within the indicated bounds of *y* = 2*x* and $y=\frac{1}{2}x$. We can also examine edges in the automated and ground-truth connectome for which the corresponding connectome has a synapse count of zero. We can, thus, see that, for all edges with a manual synapse count of at least four, we are able to recover the edge, in the sense that the automated prediction gives a synapse count of at least one. Similarly, for all edges with an automated synapse count of at least five, the edge appears in the ground-truth connectome, as the manual synapse count is at least one.

## 6. Conclusions

In this paper, we have proposed an end to end system for automatic synapse detection in EM image data, capable of handling the polyadic synapses found in *Drosophila*. We have additionally proposed a set of metrics to better assess the quality of a set of synapse predictions and whether such predictions are sufficiently accurate to be of use in connectomic studies. We evaluate our system on the *Drosophila* seven column medulla data set and show that it is capable of reconstructing high multiplicity synaptic connections, preserving biological pathways, while only making a small number of clear errors; we also show that our system greatly outperforms the baseline using body proximity as a proxy for synaptic connections.

By performing an evaluation on the entire end to end automatic predictions, we are able to assess both how each component contributes to the overall performance as well as how the components interact. For instance, by comparing performance of PSD prediction using ground-truth T-bars or ground-truth segmentation, we can estimate the expected gains from improving T-bar prediction or segmentation. At the same time, we are able to see that overall performance may be improved by taking into account noise in a previous component, such as the need to spatially shift the synapse predictions to be more robust to noise in the segmentation.

Although our proposed method is designed for synapse detection in *Drosophila*, we believe that the success of our presynaptic T-bar detector suggests that our weakly-supervised approach may be of value in other domains as well. For example, in synapse detection in mammalian tissues, rather than spending manual annotation time to obtain ground-truth labeling of synapses accurate at a voxel-level, as is common practice (Roncal et al., 2015), it may instead suffice to place two landmarks per synapse (to indicate location and directionality), allowing one to obtain more synapse annotations in an equal amount of time. Training on a larger, potentially more diverse set of synapses may lead to better accuracy and generalization across a large volume. A combination of the two approaches could also be used, wherein a small number of synapses is labeled at voxel-wise accuracy and a large number is labeled with landmarks, thereby, maintaining voxel-level performance with the added benefit of a larger, more diverse training set.

## Author contributions

GH developed and wrote core methodology, LS provided core connectome data and analysis, SP provided automated segmentation.

### Conflict of interest statement

The authors declare that the research was conducted in the absence of any commercial or financial relationships that could be construed as a potential conflict of interest.

## Footnotes

^1^https://github.com/janelia-flyem/flypylib

^2^https://www.janelia.org/project-team/flyem/data-and-software-release
